# Holobiont Urbanism: sampling urban beehives reveals cities’ metagenomes

**DOI:** 10.1186/s40793-023-00467-z

**Published:** 2023-03-30

**Authors:** Elizabeth Hénaff, Devora Najjar, Miguel Perez, Regina Flores, Christopher Woebken, Christopher E. Mason, Kevin Slavin

**Affiliations:** 1 NYU Tandon School of Engineering, Brooklyn, NY USA; 2 Center for Urban Science and Progress, NYU, Brooklyn, NY USA; 3 MIT Media Lab, Cambridge, MA USA; 4 Parsons School of Design, New York, NY USA; 5 Extrapolation Factory, New York, NY USA; 6 Department of Physiology and Biophysics, Weill Cornell Medicine, New York, NY USA; 7 Weill Cornell Medicine, The HRH Prince Alwaleed Bin Talal Bin Abdulaziz Alsaud Institute for Computational Biomedicine, New York, NY USA; 8 The WorldQuant Initiative for Quantitative Prediction, Weill Cornell Medicine, New York, NY USA

**Keywords:** Built environment, Metagenomics, Aerobiome, Holobiont, Honeybee

## Abstract

**Background:**

Over half of the world’s population lives in urban areas with, according to the United Nations, nearly 70% expected to live in cities by 2050. Our cities are built by and for humans, but are also complex, adaptive biological systems involving a diversity of other living species. The majority of these species are invisible and constitute the city’s microbiome. Our design decisions for the built environment shape these invisible populations, and as inhabitants we interact with them on a constant basis. A growing body of evidence shows us that human health and well-being are dependent on these interactions. Indeed, multicellular organisms owe meaningful aspects of their development and phenotype to interactions with the microorganisms—bacteria or fungi—with which they live in continual exchange and symbiosis. Therefore, it is meaningful to establish microbial maps of the cities we inhabit. While the processing and sequencing of environmental microbiome samples can be high-throughput, gathering samples is still labor and time intensive, and can require mobilizing large numbers of volunteers to get a snapshot of the microbial landscape of a city.

**Results:**

Here we postulate that honeybees may be effective collaborators in gathering samples of urban microbiota, as they forage daily within a 2-mile radius of their hive. We describe the results of a pilot study conducted with three rooftop beehives in Brooklyn, NY, where we evaluated the potential of various hive materials (honey, debris, hive swabs, bee bodies) to reveal information as to the surrounding metagenomic landscape, and where we conclude that the bee debris are the richest substrate. Based on these results, we profiled 4 additional cities through collected hive debris: Sydney, Melbourne, Venice and Tokyo. We show that each city displays a unique metagenomic profile as seen by honeybees. These profiles yield information relevant to hive health such as known bee symbionts and pathogens. Additionally, we show that this method can be used for human pathogen surveillance, with a proof-of-concept example in which we recover the majority of virulence factor genes for *Rickettsia felis*, a pathogen known to be responsible for “cat scratch fever”.

**Conclusions:**

We show that this method yields information relevant to hive health and human health, providing a strategy to monitor environmental microbiomes on a city scale. Here we present the results of this study, and discuss them in terms of architectural implications, as well as the potential of this method for epidemic surveillance.

**Supplementary Information:**

The online version contains supplementary material available at 10.1186/s40793-023-00467-z.

## Introduction

Over half of the world’s human population lives in urban areas and, according to the United Nations (UN), nearly 70% of us will live in cities by 2050 [1]. Our cities are built by and for humans, but are also complex, adaptive biological systems involving a diversity of living species [2]. The majority of these species are invisible and constitute the city’s microbiome. Our design decisions for the built environment shape these invisible populations, and we interact with them on a constant basis [3, 4]. A growing body of evidence shows us that our health and well-being are dependent on these interactions [5]. Indeed, multicellular organisms owe meaningful aspects of their development and phenotype to interactions with the microorganisms—bacteria or fungi—with which they live in symbiosis [6, 7]. Accumulated evidence confirms that mammalian phenotypes are related to a combination of an individual’s genotype as well as that of its microbiota, including disease states such as obesity [8] and influence on neuro-psychiatric disorders as well [9]. Beyond human consequences, plants’ flowering time has been found to depend on the soil microbiome [10] and the useful metabolic compounds in medicinal plants are possibly synthesized in conjunction with their symbiont bacteria [11], both traits formerly thought to depend only on the plant’s genotype. Metagenomic studies such as these are facilitated by the rapidly decreasing cost of high-throughput DNA sequencing, and support a growing understanding that the phenotype of a multicellular organism depends on both its own genotype and that of its associated microbes. As capacity for gathering and analyzing genomic and metagenomic data grows, our capacity to understand interspecies relationships is growing alongside it, with the potential of elucidating fundamental biological questions of host-symbiont selection and evolution mechanisms such as testing hologenome [12, 13] theories of evolution.

Metagenomics is a rapidly growing field that is well-situated to survey across all domains and kingdoms of life, including city-scale efforts of urban metagenomics. Microbial classification using high-throughput DNA sequencing is faster and more comprehensive than culture-based methods, and has enabled city-wide mapping of microbial populations [14–16]. Mapping indoor environments [3, 17] also provides insights into the relationship between humans and the indoor microbiome, which holds promise for designing buildings that optimize this metric. Thus, we are moving away from the germ-centric paradigm of microbes to the quantification of a ubiquitous, continuous and commensal map of the environmental microbiome within which we live, work, and sleep. While the processing and sequencing of samples can be high-throughput (with automation, hundreds at a time), gathering samples is still very expensive, labor intensive, and can require mobilizing large numbers of volunteers to get a snapshot of the microbial landscape of a city, such as global City Sampling Day (metasub.org). Moreover, samples collected manually with swabs represent a limited area: 0.1–0.5m^2^. While this scale of resolution is important for applications such as tracking contamination through a hospital, it is not always easily implemented for city-scale studies and leads researchers to look for pinch points where samples might be most meaningful. Examples of this have been MetaSub sampling subways [16], air sampling in indoor environments [18], or sewers [19, 20].

Setting out to collect a more distributed and comprehensive sample of the urban landscape, following conversations with artists Timo Arnall and Jack Schulze, we investigated the potential of using honeybees as proxy sampling mechanisms for the urban microbiome. On average, honeybees forage within a 1–2 mile radius around their hive in rural environments [21] and 0.3–1 miles in urban environments [22], and we hypothesized that their travel would permit them to interact with various microbial environments including air, water, and mammalian sources in addition to their known plant targets. We designed a pilot study to test for geo-specific microbial residues corresponding to all of these environments within material found in a hive.

Here we describe the results of a pilot study conducted with three rooftop beehives in Brooklyn, NY, where we evaluated the potential of various hive materials (honey, debris, hive swabs, bee bodies) to reveal information as to the surrounding metagenomic landscape, and where we conclude that the hive debris are the richest substrate. Based on these results, we profiled four additional cities by collecting hive debris: Sydney, Melbourne, Venice and Tokyo. Here we present the results of this study, and discuss them in terms of architectural implications, as well as the potential of this method for epidemic surveillance.

## Methods

### Hives and collection methods

#### U.S.A.—Brooklyn

The hives of three independent beekeepers were sampled in New York City. The first location (AS) were Langstroth hives located in Astoria, Queens, NY. The second location (CH) were Langstroth and Top Bar hives located in Crown Heights, Brooklyn, NY. The third location (FG) were Langstroth hives located in Fort Greene, Brooklyn, NY. Samples of honey, bees, hive debris, and swabs of the inside of the hive were collected using sterile one-time-use scrapers and transferred into sterile 50 ml Falcon tubes. Bee bodies were submerged in isopropyl alcohol for storage.

#### Australia—Sydney and Melbourne

Hive debris from two Langstroth hives in Sydney (SYD1, SYD2) and two in Melbourne (MEL, SH) were sampled. Custom collection trays with self-sealing apertures, designed to be placed under the hives to collect hive debris, were developed and fabricated at MIT, and shipped to Sydney and Melbourne for deployment. Trays were installed for 1 week collections, then removed and hive debris samples were transferred to sterile 50ml Falcon tubes.

#### Italy—Venice

Hive debris from one Langstroth hive at the Palazzo Mora, Venice, Italy was sampled. Debris were collected from the hive using a sterile one-time-use scraper and transferred to 50ml Falcon tube.

#### Japan—Tokyo

Hive debris amples were collected from 12 hives distributed over 4 neighborhoods. Samples were collected with sterile one-time-use scrapers and stored in sterile 50ml Falcon tubes. The locations were Marunouchi (MA), 丸の内 千代田區東京 100-0005, Mita (MI), 港區東京 108-0073日本, Marronnier Gate (MR), マロニエゲート銀座1, and Ginza (GK), 銀座 中央區東京 104-0061.

### Sample preparation

The general approach to DNA extraction involved a combination of lysis methods including mechanical, thermal, and enzymatic disruption to try and ensure that DNA from plant, microbe, and human sources would be extracted for sequencing.

#### Honey

The honey samples were diluted in a 1:1 ratio of grams of honey to mL of ultrapure water and then vortexed vigorously. The mixture was then spun down in the centrifuge at 3900 RCF for 20 minutes, the supernatant was discarded and the pellet along with

~ 200 µL residual liquid was moved to an Eppendorf, and placed in the − 20 °C freezer until the DNA extraction step.

#### Bee debris

The bee debris was diluted in a 1:5 ratio of grams of bee debris to mL of ultrapure water. The mixture was then heated in a water bath at 70 °C for 5 minutes in order to soften the debris and have it disperse in the liquid and then spun on the vortex vigorously. The liquid and solids were then separated, and both were placed into Eppendorfs and placed in the − 20 °C freezer so that a freeze-thaw cycle would help disrupt the cell membranes. The bee debris material was then ground with a mortar and pestle to break down any large pieces of bee debris, and resuspended in 1X PBS to bring all of the tubes to a final volume of 20 mL. Then material was then allowed to settle, spun down at 3900 RCF for 20 minutes along with 1–2 grams of 100µm glass beads to further mechanically disrupt the samples. The pellet and a small amount of the supernatant was then used for DNA extraction.

#### Bees

The isopropyl alcohol was drained from the tubes, then bees were placed in a mortar and pestle that was pre-chilled to − 80 °C before use. The bees were crushed vigorously into a paste. The paste was then placed in Eppendorf tubes and placed in the − 20 °C freezer until the DNA extraction step.

#### Swabs

The swabs, Copan Liquid Amies Elution Swab 481C, were stored in the − 20 °C freezer until the DNA extraction step.

### DNA extraction

The protocol for 3-5 mL of starting material of the Promega Wizard® Genomic DNA Purification Kit (A1120) was used, with the following alterations to the standard protocol: one hour incubation at 37 °C in a shaker after the neutralization step; the samples were vortexed vigorously for about 1–2 minutes after the lysis and neutralization buffer were added to mechanically disturb the material; following this a phenol/chloroform step was done to remove any remaining organic matter before being placed in the spin column; the DNA was eluted with 20 uL of TE buffer warmed to 65 °C; there was a 2 minute incubation time at room temperature before spinning down.

### Library preparation

The Library preparation protocol was performed at the Mason Lab at Weill Cornell Medicine, using the following kits according to manufacturer’s instructions. It was used to prepare libraries for all samples.

Illumina/Qiagen 500bp Prep:

1. Size selection with Agencourt AMPure XP Beads (A63881)
2. End repair and A-tailing: Qiagen GeneRead DNA Library I Core Kit (180,432)
3. Amplification: Qiagen GeneRead DNA Library I Amp Kit (180,455)
4. Illumina TruSeq DNA LT adapter kits A and B for up to 24-plex per sequencing pool.

### Sequencing

Brooklyn Pilot Study: The samples were sequenced at the BioMicro Center at MIT. The sequencing requested was a 150bp paired end sequence on one lane of the Illumina MiSeq. Venice Study: The sample was sequenced at the CNAG supercomputing center in Barcelona, Spain, with 150bp paired end reads on a Illumina MiSeq lane. Australia and Tokyo samples: Sequencing was performed on the Illumina HiSeq platform at Weill Cornell Medicine, with 125bp paired-end reads. See Additional file 6: Table S1 for read counts for all samples.

## Analysis

### Metagenomic classification

Read quality was assessed with FastQC [23] and read quality was sufficient to not require trimming (see Additional file 7 for sample metadata, and Additional file 8 for MetaQC [24] reports). DIAMOND [25] – MEGAN [26] against the NCBI-nr database was used for read classification, as described in [27].

run diamond:

for file in *.fastq.gz; do name=${file/.fastq.gz/}; diamond blastx

-d /path/to/NCBI_nr/nr -q $file -a $name -p 16

convert binary DIAMOND format to BLAST tabular format:

for file in *.daa; do diamond view --daa $file --out

${file/.daa/}.tab --outfmt tab; echo $file; done

perform read-by-read taxonomy classification with MEGAN:

for file in *.tab; do /path/to/programs/megan/tools/blast2lca -- input $file --format BlastTAB --topPercent 10 --gi2taxa

/path/to/programs/megan/GI_Tax_mapping/gi_taxid-March2015X.bin-- output $file.read_assignments.txt; done

Heatmaps were generated with the script metaphlan_hclust_heatmap.py from the MetaPhlan package, displaying the abundances for species only (default –tax_lev s), in logarithmic scale (-s log). The clustering is performed with "average" linkage (default -m average), using "Bray–Curtis" distance for clades (default -d braycurtis) and "correlation" for samples (default -f correlation).

metaphlan_hclust_heatmap.py –in $file –out $file.Blues.minv0.maxv1.Blues.log.pdf -c Blues -s log −minv 0.0 –maxv 1.

### Diversity quantification

Beta-diversity was calculated according to the Bray-Curtis dissimilarity metric (Bray and Curtis 1957) as implemented by the Qiime2 package [28].

$ metaphlan2biom.py merged.samples.metaphlan.out merged.samples.biom

$ beta_diversity.py -i merged.samples.biom -m bray_curtis -o merged.samples.beta_div.bray_curtis

*P*-value was calculated based on 100 bootstrapped subsamples of the Brooklyn debris sample, each subsample being of 1 million reads. Bootstrapped samples were classified using the same methods as described above, and pairwise beta-diversity calculated as above. *P*-value was calculated as the number of bootstrap samples with lesser dissimilarity value than the test value.

### Assembly and contig annotation

Co-assembly of Tokyo samples (assembly of all sequences pooled together) was performed with MegaHit [29] and reads for each individual sample were mapped to contigs with Bowtie2 [30]. Assembly yielded 3207501 contigs with a total of 2802811167 base pairs. Contig length ranged from 200 to 488034 base pairs, with an average of 874bp and an N50 of 1515bp. Contigs were annotated with Anvio [23].

### Virulence factor identification

Virulence factors for *Rickettsia felis* were downloaded from the Virulence Factors of Pathogenic Bacteria database http://www.mgc.ac.cn/cgi-bin/VFs/compvfs.cgi). BLAST [31] was used to align the virulence factor genes to the assembled contigs, reporting the query coverage and percent identity.

## Results

### Brooklyn pilot study

**Fig. 1:**
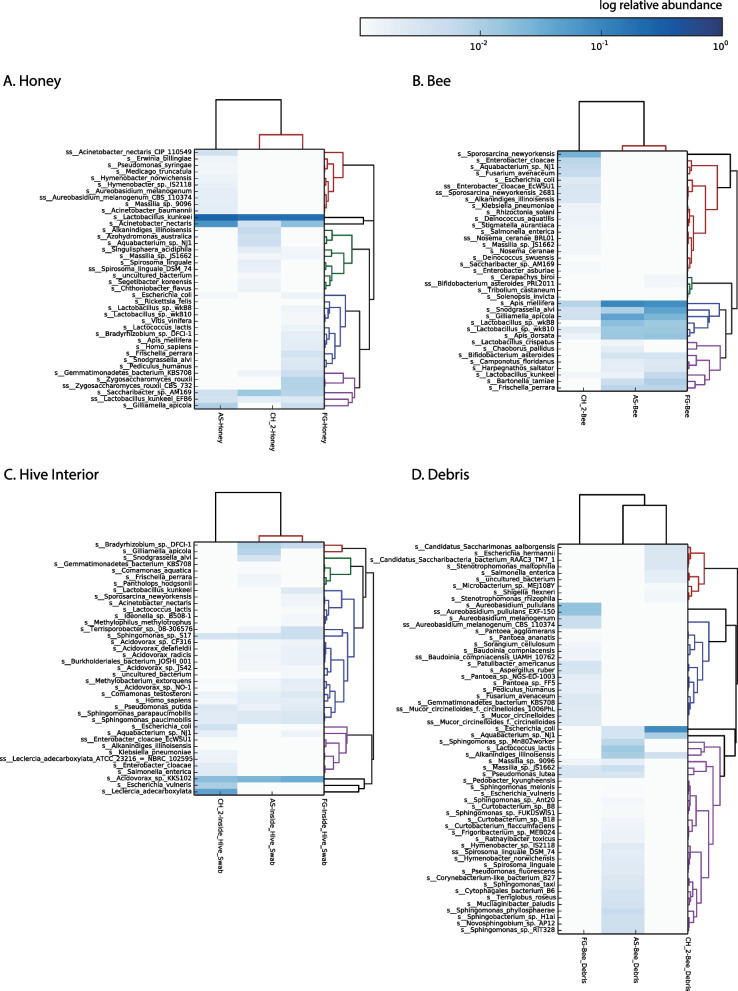
Species classification by type of sample in Brooklyn pilot study: **A** Honey, **B** Bee body, **C** Hive interior, **D** Debris. Hives are abbreviated as: *AS* Astoria, *CH* Crown Heights, *FG* Fort Greene. Color map scale corresponds to the log of relative abundance in each sample

In order to assess the potential of using honeybees as metagenomic “sample collectors”, we designed a pilot study with three Langstroth hives in Brooklyn, wherein we sampled the interior of the hive, the debris at the bottom, bee bodies, and honey. We sequenced the DNA of each sample using a high-throughput shotgun approach, and classified the reads using DIAMOND-MEGAN against the NCBI NR nucleotide database, which includes all kingdoms and domains of life (see Methods for more details) (Fig. 1). The honey of each hive is largely dominated by the species *Lactobacillus kunkeei* (Fig. 1A), an obligate fructophilic lactic acid bacteria found in flowers, wine, and honey [32]. Also of note are *Acinetobacter nectaris*, found in flowers [33], and *Zygosaccharomyces rouxii*, known to thrive under salt or sugar osmotic stress and thus cause food spoilage [34]. Bee gut commensals were found in low abundance in honey, and include the species identified in the bee body samples, described below. Traces of plant DNA were also identified, including *Medicago truncatula* and *Vitis vinifera*. The bee body samples (Fig. 1B) contain sequences representative of both *Apis mellifera* (European honeybee) and *Apis dorsata* (Giant honeybee), indicating the hives are likely hybrids of these two species. The most abundant microbes in the bee body samples include species described as bee commensals such as *Snodgrassella alvi* and *Gilliamella apicola* [35], as well as *Lactobacillus wkB8* and *wkB10* [34]. The bees from AS and FG hives display almost identical species distribution, however the bees from the CH hive show lower abundances of the aforementioned commensals, and present species absent from the other two. These include *Nosema ceranae*, a fungal parasite of the honeybee affecting both larvae and adults [37], as well as various human-related bacteria such as *Sporosarcina newyorkensis*, isolated from clinical samples in New York State [38] and *Enterobacter* species. We hypothesize the colonization of atypical bacteria in this bee is correlated to the dysbiosis caused by *Nosema* infection.

The inside of the hives (Fig. 1C) was quite uniform across locations, and dominated by environmental bacterial species usually described as found in polluted environments. These include *Acidovorax sp. KKS102*, known to degrade biphenyl/polychlorinated biphenyls (PCBs) [39], *Sphingomonas sp. S17* [40], found in high-altitude Andean lakes and tolerant to high pH and desiccation. The interior of beehives is coated with propolis, a resinous substance including polyphenols from essential oils and with a pH of 8.5 [41]. It is a strong antimicrobial, antifungal and antiviral agent [42] and therefore we hypothesize the presence of extremophile bacteria, and their similar distribution across hives, is a result of selection by the chemical properties of propolis. The species identified in the debris samples (Fig. 1 C) were the most diverse (Table 1), and include several species of plants as well as plant-associated microbes such at the fungus *Aureobasium pullulans,* also an opportunistic human pathogen [43], aquatic microbes such as the alkane-degrading *Aquabacterium sp. NJ1* [44] and honeybee associated such as *Stenotrophomonas maltophilia* [45] (also known as an opportunistic mammalian pathogen [46]). Taken together, the samples cluster according to sample type, versus sample location (Additional file 1: Fig S1). As a control, we also sampled a beekeeper’s hands and hive scraper tool (in one instance) as well as the hive exterior, and these samples were notably different than the debris as well (Additional file 1: Fig S1). The former control indicates that the signatures in the debris collected are not just from manipulation, and the latter indicates that the debris composition is not just from settling of material from the environment immediately exterior to the hive.

**Table 1:**
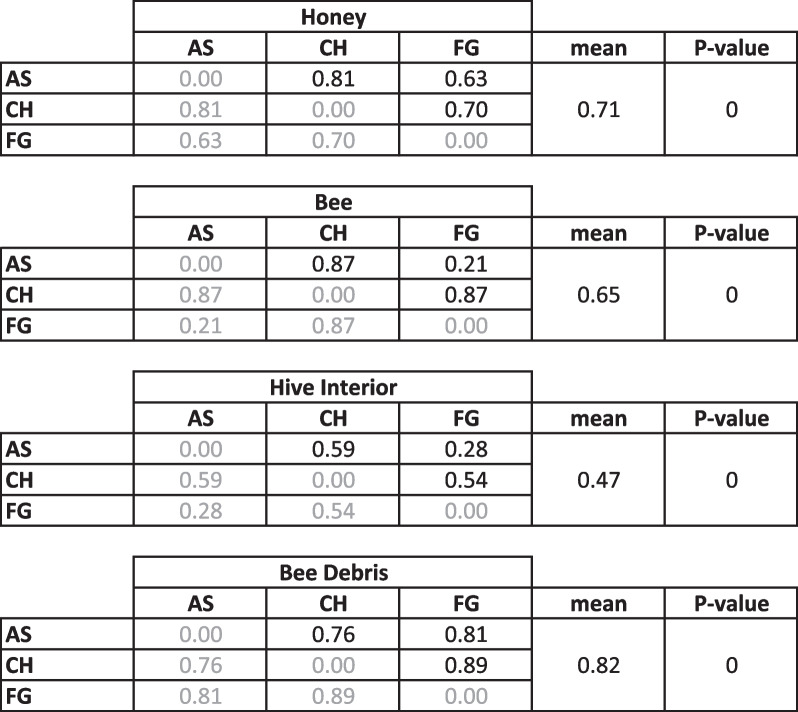
Beta-diversity according to sample type (Bray–Curtis dissimilarity)

*P*-value calculated against 100 random subsamples of a debris sample. Hives are abbreviated as: *AS* Astoria, *CH* Crown Heights, *FG* Fort Greene

While samples from different hives within a sample type are significantly different from each other (*P* = 0.0) according to Bray–Curtis dissimilarity (Table 1), we found the debris samples to be the most diverse, as well as have the highest proportion of environmental bacteria. As our interest was to collect metagenomic information of the environment the bees traverse, rather than that of their hive, we concluded that bee debris is the best material for that purpose.

### Urban metagenomes as seen by bees

**Fig. 2:**
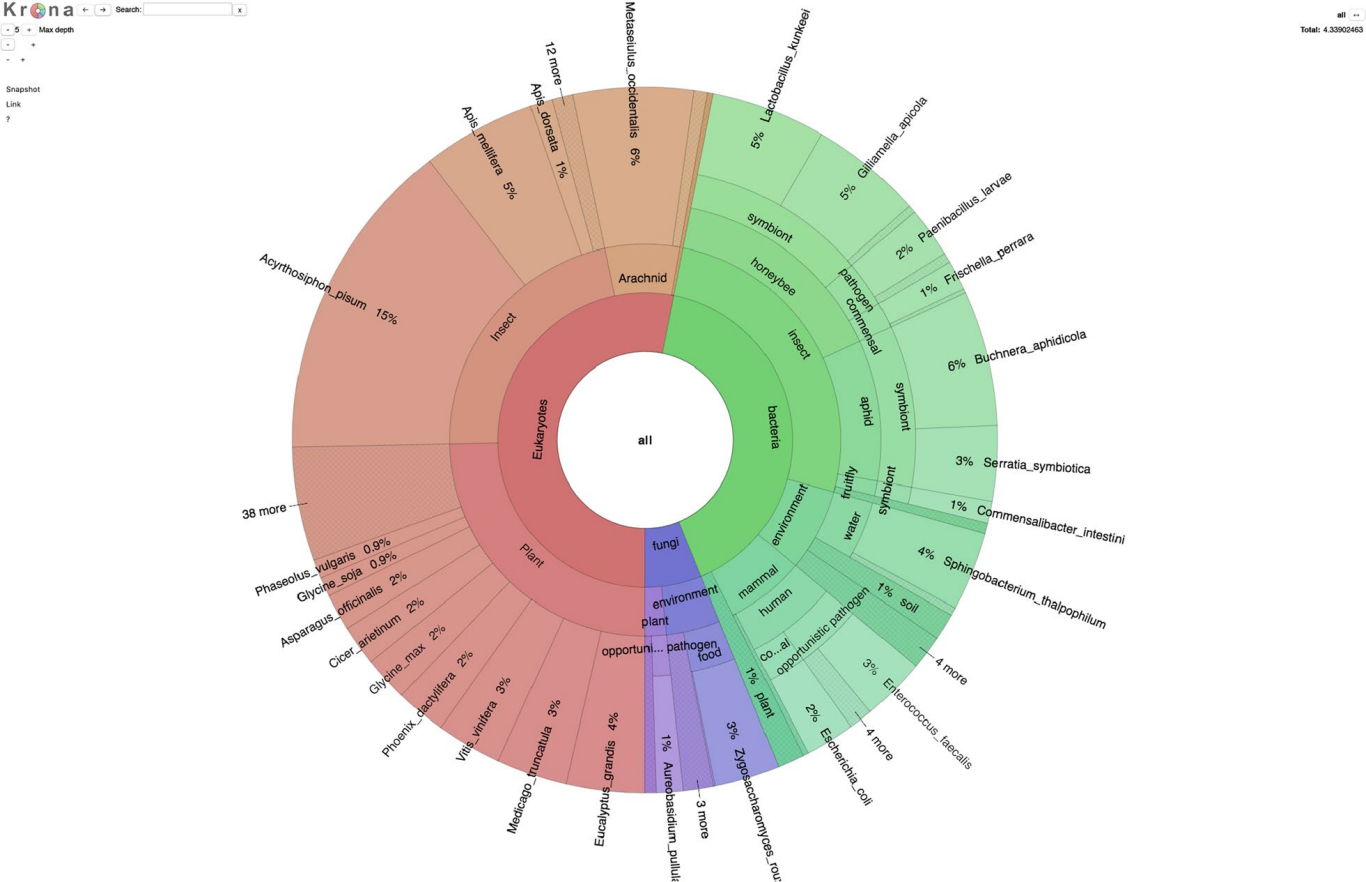
Distribution among kingdoms of classified reads across all samples, including most abundant species in each category

We next sampled bee hive debris from four cities across the world: Venice, Italy; Sydney and Melbourne in Australia; several neighborhoods in Tokyo, Japan. Over all of these locations, we recovered DNA from plants, mammals, insects, arachnids, bacteria and fungi. Taken together, 53% of the classified reads were from multicellular organisms, and 47% from microorganisms. (Fig 2).

All metagenomes characterized show different signatures according to cities (Additional file 2: Fig S2), and have particularities that can be related to the identity of the city. The metagenome of the debris collected from the hive in Venice was largely dominated by fungi related to wood rot (Additional file 3: Fig S3), which is a common feature of the buildings, built on submerged wooden pilings, and date palm DNA. Melbourne’s sample was dominated by Eucalyptus DNA, while Sydney’s showed little plant DNA, but bacteria such as *Gordonia polyisoprenivorans*, which degrades rubber[47] (Additional file 4: Fig S4). Tokyo’s metagenome includes plant DNA from Lotus and wild soybean, as well as the soy sauce fermenting yeast *Zygosaccharomyces rouxii* [34] (Additional file 5: Fig S5). Overall, each city has a unique metagenomic signature as viewed by bees, with microbes coming from a variety of sources: environmental, insect-related, mammalian and aquatic (see Table 2 for relative abundances of bacteria associated with different hosts or environments).

**Table 2:**
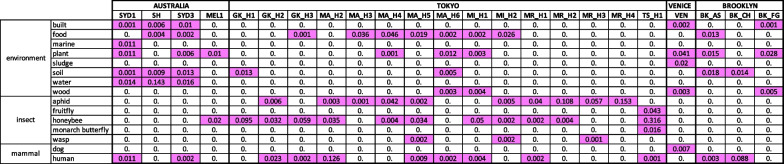
Major classes of bacteria across samples

This table summarizes the most abundant bacterial species (accounting for 90% of bacterial contribution to a sample’s profile) according to their associated host or environment. Numbers are relative abundance (normalized to 1 for each sample). Colored cells indicate values over 0

### Debris as indicator of hive health

As the debris include parts of bees, we looked to the data to see if we could find microbes related to bee health. We found three honey and bee crop related species such as *Lactobacillus kunkeii*, *Saccharibacter sp. AM169 and Frishella perrara* and five bee gut species, with Gilliamella apicola being found in the most samples (Table 3)[48]. We also identified known bee pathogens, namely *Paenibacillus larvae* and *Melissococcus plutonius,* as well as the parasite *Varroa destructor*. These results indicate that debris may be used to assess overall hive health, or to assess the interaction of bee related species with environmental microbial species.

**Table 3:**
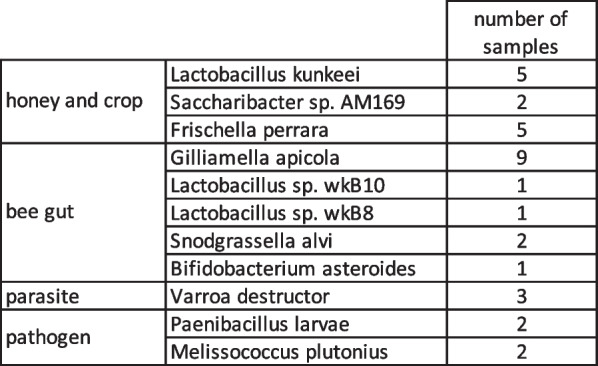
Bee related species: known bee gut species, honey and bee crop species, pathogens, and parasites

### Debris as indicator of human health

As the bees are traversing densely populated urban areas, we tested the hypothesis that they may be able to recover human pathogens and assess their pathogenic capacity by identifying virulence factor genes. Virulence factors are the molecules that enable the specific pathogenicity of the micro-organism [49]. Given the high level of genomic variation within species, asserting the presence of a pathogen through taxonomic classification is not sufficient to assert its pathogenicity. For this, we proceeded by performing de-novo co-assembly of the sequences from a given city, then using a metagenomic-specific classifier targeted to identify bacterial species from the contigs. We identified various opportunistic pathogens as well as some known disease-causing pathogens, including *Shigella dysenteriae Sd197* (causing bacillary dysentry [50]) and *Rickettsia felis* (causing “cat scratch fever” [51]). We selected the Tokyo dataset for assembly as this location presented the highest number of samples, samples collected at two timepoints, as well as highest sequencing coverage per sample. We chose *Rickettsia felis* as an example to demonstrate the ability to identify a pathogen and its virulence factors with this sample collection method as it was the most represented in the assembled contigs. To go beyond species classification and assess pathogenic potential, we queried the assembled metagenome for *Rickettsia felis* virulence factor genes, as their presence is required for pathogenic capacity. We used *R. felis* as a proof-of-principle example that it is possible to verify pathogenic capacity of classified species with this type of data. In the Tokyo dataset, we recovered 28 of the 31 *Rickettsia felis* virulence genes with high coverage and at high similarity on the nucleotide level (Table 4). While co-assembly of these complex metagenomes led to less than optimal N50 values (N50=1515bp), this assembly quality was sufficient for virulence factor gene identification, as the genes tested for *Rickettsia felis* were covered over 97% of their length on average (Table 5) when aligned to the assembled contigs.

**Table 4:**
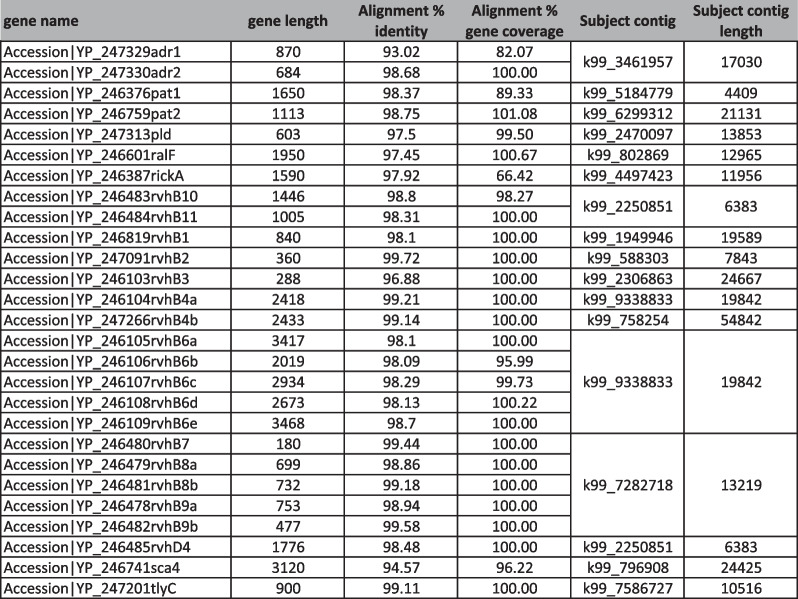
Alignment statistics of Rickettsia felis virulence factor genes mapped to assembled contigs of Tokyo metagenome

**Table 5:**
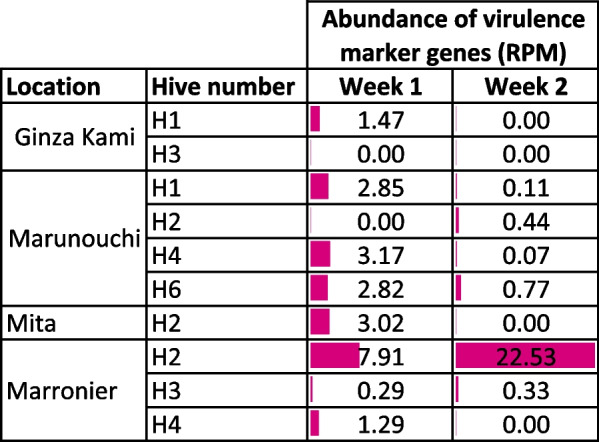
Abundance of virulence factors in samples collected at 1-week interval in Tokyo

Numbers reported are Reads per Million. Colored bars represent numerical values of each cell

We assessed the persistence of virulence factors in the debris by analyzing samples taken at a 1- week interval in the Tokyo hives. After the first sampling, the bottom trays were cleaned and debris was collected after a week. In some cases, no markers were observed in the second samples, indicating that the cleaning was effective. In the Marunouchi hive H2, markers were found again, and more abundantly (Table 5). This indicates virulence markers that are either very abundant in the bee’s range or that they can change rapidly in abundance.

## Discussion

Here we show that honeybees are relevant sensors for the urban microbiome, and that the debris collected contain a trace of the microbial clouds the bees are traversing as well as carry indicators of hive health. While these methods are cost prohibitive for amateur or even professional beekeepers as pathogen detection, and existing targeted methods already exist, these results present a methodology to assess additional dimensions of hive health. Indeed, we show that bees interact with a wide range of microbial species and thus future apiculture research could consider individual hive health in relation to the bees’ microbial environment, exploiting for example existing databases and scripts describing bee-associated bacteria [52]. Indeed, these bees recover microbes associated with plants, with which they have physical interactions, but also of mammals and aquatic environments, with which they presumably do not have direct contact. This implies that these microbes were constituents of the respective “microbial clouds” [53] of these entities and that the bees collect a trace of these clouds. Biological content in the atmosphere—the biosphere—was first described in 1978 [54] and has since been characterized as an integral part of ecosystem function [55]. The biosphere is an indicator of climate change, for example, increasing frequency of dust storms from the African continent are carrying plant and aquatic pathogens to the Americas, affecting coral populations [56]. Urban aerosols contain a diverse microbial component including species of potential health and bioterrorism concern. This study demonstrates a novel sampling methodology, with consistent results with a recent study using shotgun sequencing of honey to assess bee core gut microbiomes as well as plant species interaction while foraging [57], while also providing additional environmental microbiome data than the honey substrate. This reveals that different neighborhoods have different clouds just as different humans do, and that the collected microbiome can reveal information about the built environment and its inhabitants. For example, the Venetian bees carried a signature of wood rot and aquatic species, similar to previous work showing how flooded areas of a city can carry a “molecular echo” of the aquatic events of its past [14]. Indeed, it has been shown that microbial communities can serve as quantitative geochemical indicators [58] and the metabolic properties of the recovered communities can yield information about the environment. Furthermore, metagenomic data can be mined for human-health related information [59]. Future uses of data collected in this manner could be assessment of antibiotic resistance gene profiles, and while the molecular and computational methods used here were based on DNA analysis, it is possible they could be used to monitor RNA-based viruses such as Sars-Cov-2 or other future airborne pathogens, as demonstrated by targeted analyses using swab-based collection at hive doors during the COVID19 global pandemic [60].

## Conclusions

Our ability to recover virulence factors associated with human disease indicates that this method can serve for early detection of human-associated pathogens, in a complimentary modality to existing biosurveillance methods such as indoor air or sewage monitoring. However, this multi-species methodological approach may hold even more hope for a diversified understanding of urban microbiomes, their relationship to the built environment, and their relationship to human and other non-human species. Indeed, insect-based, city-wide microbial monitoring is likely more spatially comprehensive, even if lower resolution, compared to discrete, human-based sampling techniques, such as swabbing or air-sampling. This method offers the capacity to further catalog the urban environmental microbiome, contributing information to our understanding of its impact on humans. Additionally, this methodology offers a framework to understand multispecies interactions in the built environment, namely understanding hive health in the context of the microbiome of the bees’ foraging range.

We have the unique possibility to understand our built environment and therefore design it, not just for ourselves but for all its inhabitants, from environments as common and public as subways [61] to those as specialized and hermetic as space stations [62, 63]. As Jane Jacobs says, “Cities are an immense laboratory of trial and error, failure and success, in city planning and city design” [64]. Through studies such as the one presented here, and using interdisciplinary approaches including art practice [65], we aim to further understand this accidentally engineered multispecies experiment of our built, shared, environment.

## Supplementary Information

**Additional file 1.** Clustered heatmap of Brooklyn pilot samples including hive debris, bee bodies, honey, propolis, swabs of the hive structure as well as the beekeepers’ hands.

**Additional file 2.** Clustered heatmap of hive debris samples from USA, Italy, Australia and Japan.

**Additional file 3.** Heatmap of Venice (Italy) hive debris samples.

**Additional file 4.** Heatmap of Sydney and Melbourne (Australia) hive debris samples.

**Additional file 5.** Heatmap of Tokyo (Japan) hive debris samples.

**Additional file 6.** Read counts for all samples.

**Additional file 7.** Metadata for all samples in MIXS format.

**Additional file 8.** MultiQC reports for samples by country.

## Data Availability

The datasets generated during the current study are available in the National Center for Biotechnology Information under BioProject accession PRJNA630108 (https://www.ncbi.nlm.nih.gov/bioproject/PRJNA630108) and the Sequence Read Archive repository under the accession SRP259669 (https://trace.ncbi.nlm.nih.gov/Traces/sra/?study=SRP259669).
