# Supplementary material for: Holobiont Urbanism: sampling urban beehives reveals cities’ metagenomes
Source: Environ Microbiome. 2023 Mar 30;18:23. doi: 10.1186/s40793-023-00467-z (PMC10060141; doi:10.1186/s40793-023-00467-z)
Supplement: Supplementary file 1 — Additional file 1. Clustered heatmap of Brooklyn pilot samples including hive debris, bee bodies, honey, propolis, swabs of the hive structure as well as the beekeepers’ hands. [file 40793_2023_467_MOESM1_ESM.pdf]

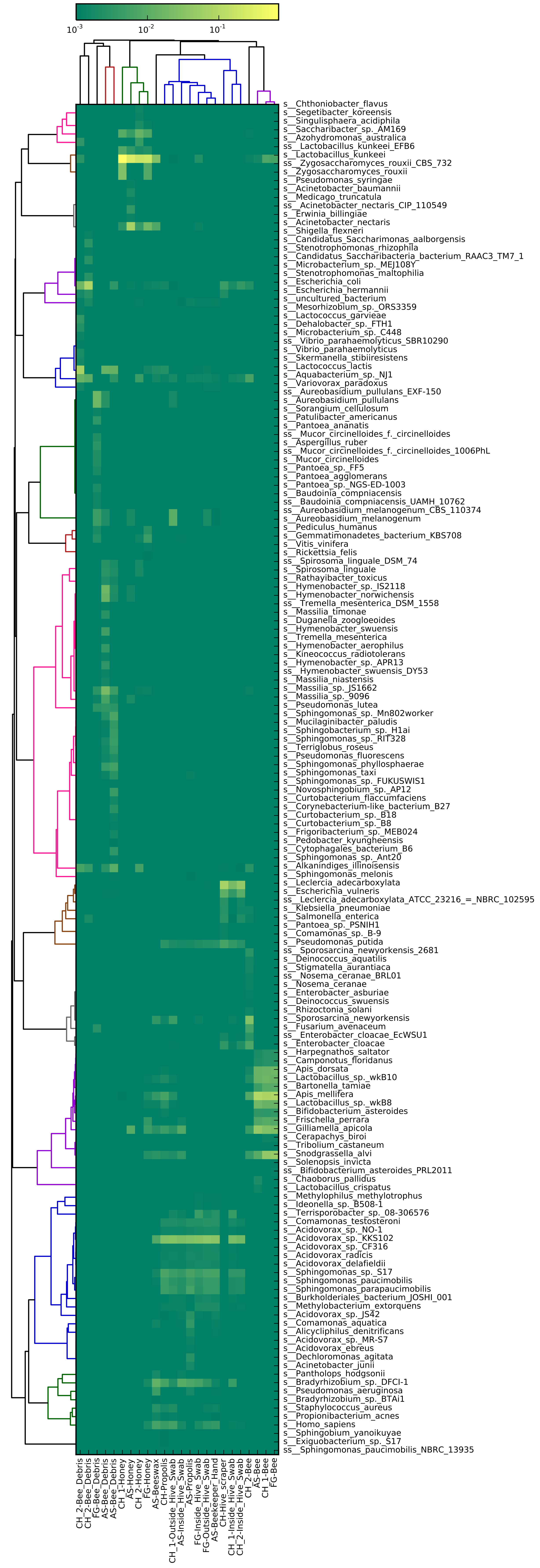

10^-3
10^-2
10^-1
s__Chthoniobacter_flavus
s__Segetibacter_koreensis
s__Singulisphaera_acidiphila
s__Saccharibacter_sp._AM169
s__Azohydromonas_australica
ss__Lactobacillus_kunkeei_EFB6
s__Lactobacillus_kunkeei
ss__Zygosaccharomyces_rouxii_CBS_732
s__Zygosaccharomyces_rouxii
s__Pseudomonas_syringae
s__Acinetobacter_baumannii
s__Medicago_truncatula
ss__Acinetobacter_nectaris_CIP_110549
s__Erwinia_billingiae
s__Acinetobacter_nectaris
s__Shigella_flexneri
s__Candidatus_Saccharimonas_aalborgensis
s__Stenotrophomonas_rhizophila
s__Candidatus_Saccharibacteria_bacterium_RAAC3_TM7_1
s__Microbacterium_sp._MEJ108Y
s__Stenotrophomonas_maltophilia
s__Escherichia_coli
s__Escherichia_hermannii
s__uncultured_bacterium
s__Mesorhizobium_sp._ORS3359
s__Lactococcus_garvieae
s__Dehalobacter_sp._FTH1
s__Microbacterium_sp._C448
ss__Vibrio_parahaemolyticus_SBR10290
s__Vibrio_parahaemolyticus
s__Skermanella_stibiiresistens
s__Lactococcus_lactis
s__Aquabacterium_sp._NJ1
s__Variovorax_paradoxus
ss__Aureobasidium_pullulans_EXF-150
s__Aureobasidium_pullulans
s__Sorangium_cellulosum
s__Patulibacter_americanus
s__Pantoea_ananatis
ss__Mucor_circinelloides_f._circinelloides
s__Aspergillus_ruber
ss__Mucor_circinelloides_f._circinelloides_1006PhL
s__Mucor_circinelloides
s__Pantoea_sp._FF5
s__Pantoea_agglomerans
s__Pantoea_sp._NGS-ED-1003
s__Baudoinia_compniacensis
ss__Baudoinia_compniacensis_UAMH_10762
ss__Aureobasidium_melanogenum_CBS_110374
s__Aureobasidium_melanogenum
s__Pediculus_humanus
s__Gemmatimonadetes_bacterium_KBS708
s__Vitis_vinifera
s__Rickettsia_felis
ss__Spirosoma_linguale_DSM_74
s__Spirosoma_linguale
s__Rathayibacter_toxicus
s__Hymenobacter_sp._IS2118
s__Hymenobacter_norwichensis
ss__Tremella_mesenterica_DSM_1558
s__Massilia_timonae
s__Duganella_zoogloeoides
s__Hymenobacter_swuensis
s__Tremella_mesenterica
s__Hymenobacter_aerophilus
s__Kineococcus_radiotolerans
s__Hymenobacter_sp._APR13
ss__Hymenobacter_swuensis_DY53
s__Massilia_niastensis
s__Massilia_sp._JS1662
s__Massilia_sp._9096
s__Pseudomonas_lutea
s__Sphingomonas_sp._Mn802worker
s__Mucilaginibacter_paludis
s__Sphingobacterium_sp._H1ai
s__Sphingomonas_sp._RIT328
s__Terriglobus_roseus
s__Pseudomonas_fluorescens
s__Sphingomonas_phyllosphaerae
s__Sphingomonas_taxi
s__Sphingomonas_sp._FUKUSWIS1
s__Novosphingobium_sp._AP12
s__Curtobacterium_flaccumfaciens
s__Corynebacterium-like_bacterium_B27
s__Curtobacterium_sp._B18
s__Curtobacterium_sp._B8
s__Frigoribacterium_sp._MEB024
s__Pedobacter_kyungheensis
s__Cytophagales_bacterium_B6
s__Sphingomonas_sp._Ant20
s__Alkanindiges_illinoisensis
s__Sphingomonas_melonis
s__Leclercia_adecarboxylata
s__Escherichia_vulneris
ss__Leclercia_adecarboxylata_ATCC_23216_=_NBRC_102595
s__Klebsiella_pneumoniae
s__Salmonella_enterica
s__Pantoea_sp._PSNIH1
s__Comamonas_sp._B-9
s__Pseudomonas_putida
ss__Sporosarcina_newyorkensis_2681
s__Deinococcus_aquatilis
s__Stigmatella_aurantiaca
ss__Nosema_ceranae_BRL01
s__Nosema_ceranae
s__Enterobacter_asburiae
s__Deinococcus_swuensis
s__Rhizoctonia_solani
s__Sporosarcina_newyorkensis
s__Fusarium_avenaceum
ss__Enterobacter_cloacae_EcWSU1
s__Enterobacter_cloacae
s__Harpegnathos_saltator
s__Camponotus_floridanus
s__Apis_dorsata
s__Lactobacillus_sp._wkB10
s__Bartonella_tamiae
s__Apis_mellifera
s__Lactobacillus_sp._wkB8
s__Bifidobacterium_asteroides
s__Frischella_perrara
s__Gilliamella_apicola
s__Cerapachys_biroi
s__Tribolium_castaneum
s__Snodgrassella_alvi
s__Solenopsis_invicta
ss__Bifidobacterium_asteroides_PRL2011
s__Chaoborus_pallidus
s__Lactobacillus_crispatus
s__Methylophilus_methylotrophus
s__Ideonella_sp._B508-1
s__Terrisporobacter_sp._08-306576
s__Comamonas_testosteroni
s__Acidovorax_sp._NO-1
s__Acidovorax_sp._KKS102
s__Acidovorax_sp._CF316
s__Acidovorax_radicis
s__Acidovorax_delafieldii
s__Sphingomonas_sp._S17
s__Sphingomonas_paucimobilis
s__Sphingomonas_parapaucimobilis
s__Burkholderiales_bacterium_JOSHI_001
s__Methylobacterium_extorquens
s__Acidovorax_sp._JS42
s__Comamonas_aquatica
s__Alicycliphilus_denitrificans
s__Acidovorax_sp._MR-S7
s__Acidovorax_ebreus
s__Dechloromonas_agitata
s__Acinetobacter_junii
s__Pantholops_hodgsonii
s__Bradyrhizobium_sp._DFCI-1
s__Pseudomonas_aeruginosa
s__Bradyrhizobium_sp._BTAi1
s__Staphylococcus_aureus
s__Propionibacterium_acnes
s__Homo_sapiens
s__Sphingobium_yanoikuyae
s__Exiguobacterium_sp._S17
ss__Sphingomonas_paucimobilis_NBRC_13935
CH_2-Bee_Debris
CH_1-Bee_Debris
FG-Bee_Debris
AS-Bee_Debris
AS-Bee_Debris
CH_1-Honey
AS-Honey
CH_2-Honey
FG-Honey
AS-Beeswax
CH-Propolis
CH_1-Outside_Hive_Swab
AS-Inside_Hive_Swab
AS-Propolis
FG-Inside_Hive_Swab
FG-Outside_Hive_Swab
AS-BeeKeeper_Hand
CH-Hive_scraper
CH_1-Inside_Hive_Swab
CH_2-Inside_Hive_Swab
CH_2-Bee
AS-Bee
CH_1-Bee
FG-Bee
