# Supplementary figures and images for: Holobiont Urbanism: sampling urban beehives reveals cities’ metagenomes
Source: Environ Microbiome. 2023 Mar 30;18:23. doi: 10.1186/s40793-023-00467-z (PMC10060141; doi:10.1186/s40793-023-00467-z)

# debris all

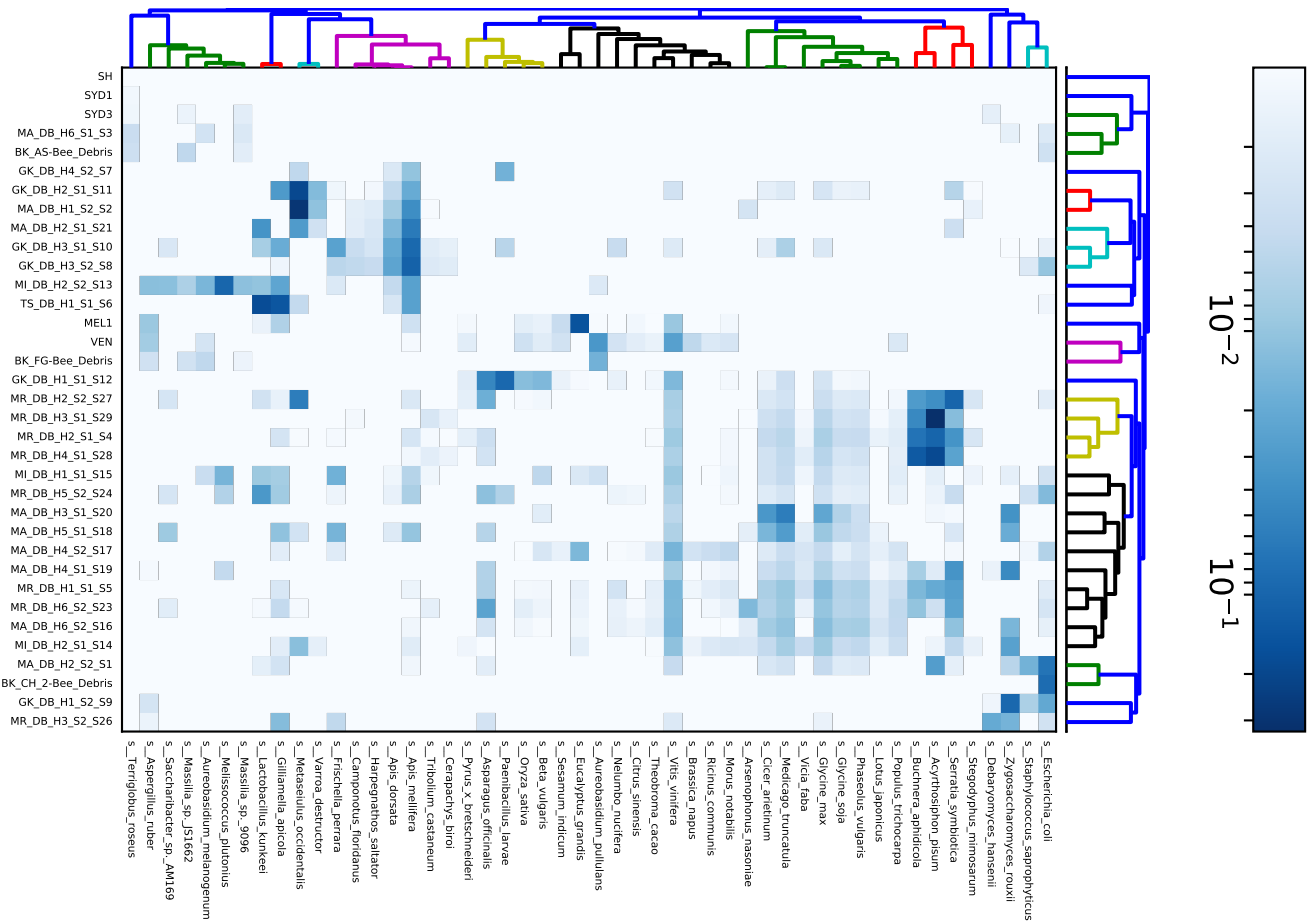

Supplement: Supplementary file 2 — Additional file 2. Clustered heatmap of hive debris samples from USA, Italy, Australia and Japan. [file 40793_2023_467_MOESM2_ESM.pdf]

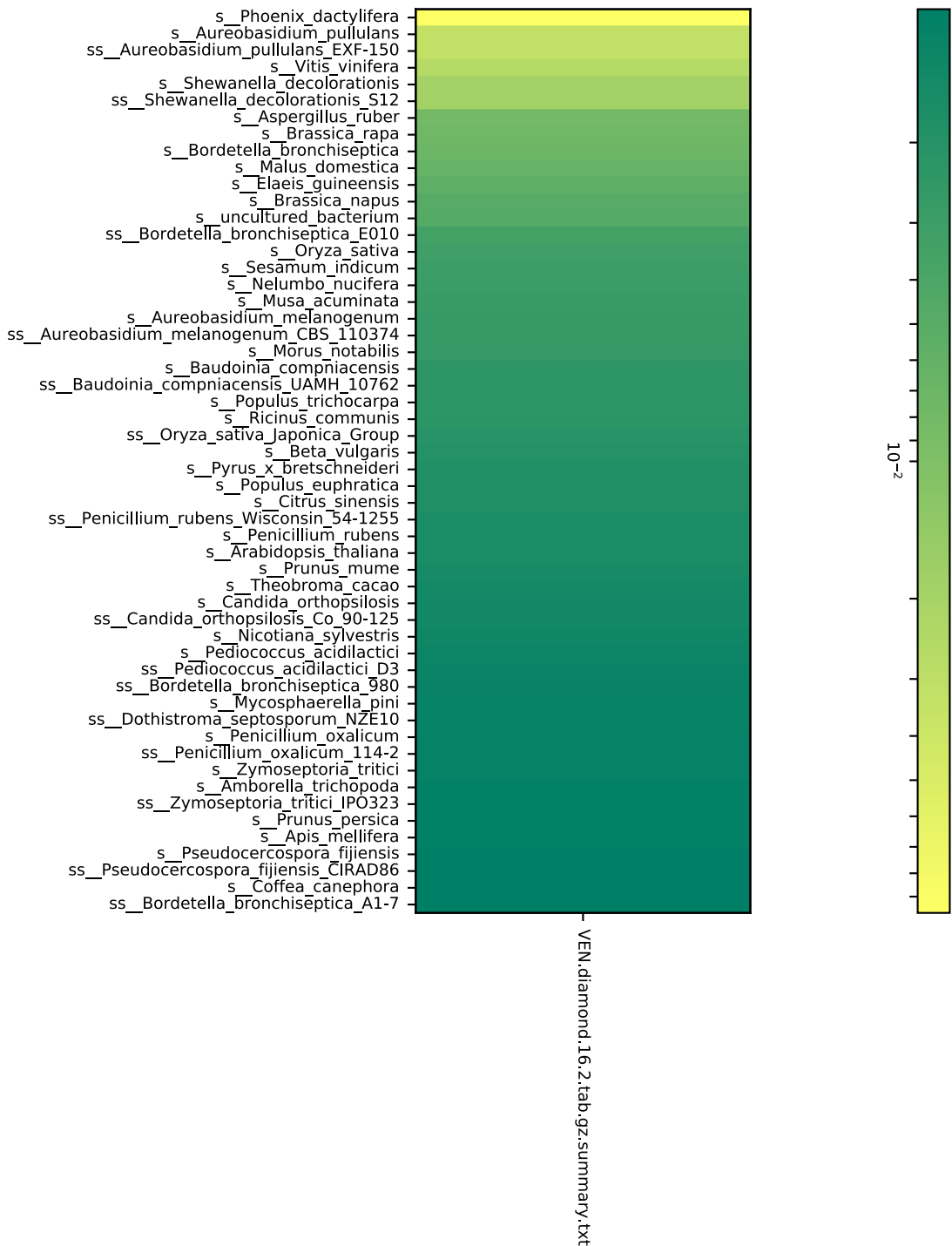

Supplement: Supplementary file 3 — Additional file 3. Heatmap of Venice (Italy) hive debris samples. [file 40793_2023_467_MOESM3_ESM.pdf]

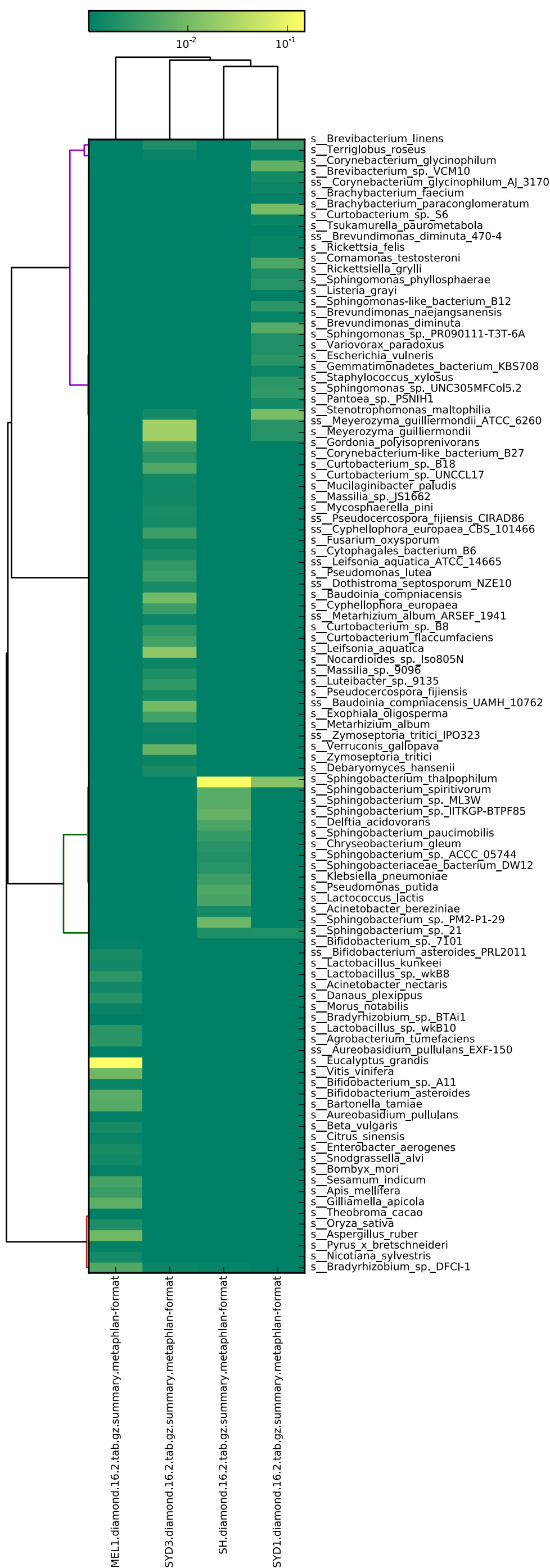

Supplement: Supplementary file 4 — Additional file 4. Heatmap of Sydney and Melbourne (Australia) hive debris samples. [file 40793_2023_467_MOESM4_ESM.pdf]

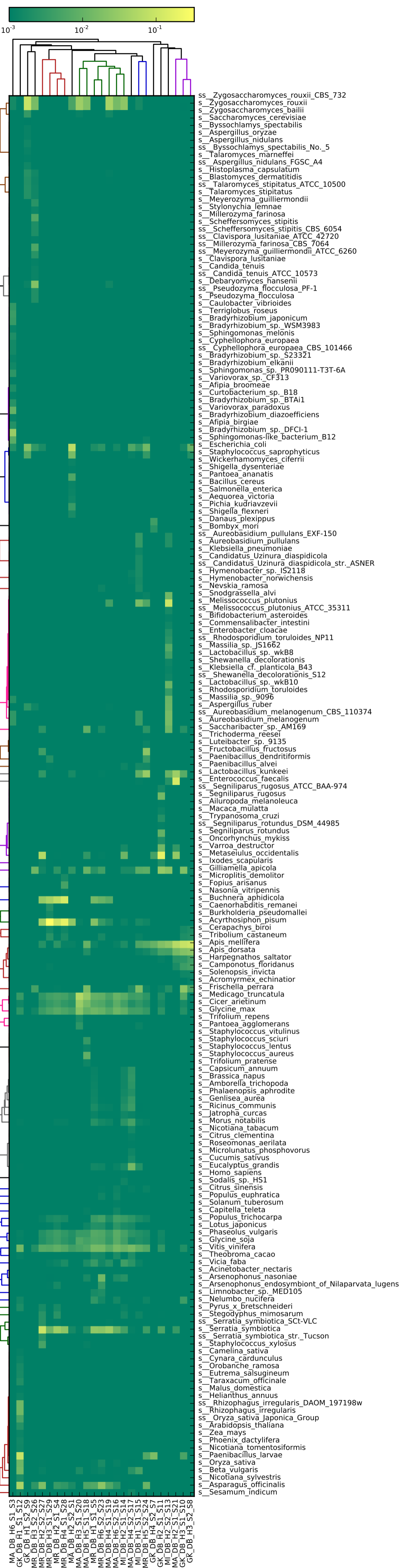

Supplement: Supplementary file 5 — Additional file 5. Heatmap of Tokyo (Japan) hive debris samples. [file 40793_2023_467_MOESM5_ESM.pdf]
