# Supplementary material for: Holobiont Urbanism: sampling urban beehives reveals cities’ metagenomes
Source: Environ Microbiome. 2023 Mar 30;18:23. doi: 10.1186/s40793-023-00467-z (PMC10060141; doi:10.1186/s40793-023-00467-z)
Supplement: Supplementary file 6 — Additional file 6. Read counts for all samples. [file 40793_2023_467_MOESM6_ESM.pdf]

Supplementary Table 1

	Sample	Million Reads
Brooklyn	AS-Bee	1.519066
	AS-Bee_Debris	1.690898
	AS-Bee_Debris	1.918464
	AS-Honey	1.754984
	AS-Inside_Hive_Swab	1.14609
	CH_2-Bee	1.823688
	CH_2-Bee_Debris	1.865624
	CH_2-Bee_Debris	1.368362
	CH_2-Honey	1.72086
	CH_2-Inside_Hive_Swab	1.282562
	FG-Bee	1.819204
	FG-Bee_Debris	1.907648
	FG-Honey	1.255524
	FG-Inside_Hive_Swab	1.487172
Tokyo	GK_H1_S1_S12	12.22419
	GK_H1_S2_S9	11.449704
	GK_H3_S1_S10	10.703352
	GK_H3_S2_S8	28.83604
	MA_H1_S1_S22	58.925544
	MA_H1_S2_S2	130.585438
	MA_H2_S1_S21	21.018622
	MA_H2_S2_S1	13.770676
	MA_H4_S1_S19	24.612872
	MA_H4_S2_S17	26.797518
	MA_H6_S1_S3	0.70942
	MA_H6_S2_S16	23.242922
	MI_H2_S1_S14	17.856454
	MI_H2_S2_S13	17.317142
	MR_H2_S1_S4	14.153042
	MR_H2_S2_S27	25.658876
	MR_H3_S1_S29	27.467216
	MR_H3_S2_S26	30.420888
	MR_H4_S1_S28	23.282586
	MR_H4_S2_S25	12.263354
Australia	MEL1	46.579378
	SH	36.062846
	SYD1	42.08362
	SYD3	45.179888
Venice	Venice	24.690872
